# Common skin bacteria protect their host from oxidative stress through secreted antioxidant RoxP

**DOI:** 10.1038/s41598-019-40471-3

**Published:** 2019-03-05

**Authors:** Tilde Andersson, Gizem Ertürk Bergdahl, Karim Saleh, Helga Magnúsdóttir, Kristian Stødkilde, Christian Brix Folsted Andersen, Katarina Lundqvist, Anders Jensen, Holger Brüggemann, Rolf Lood

**Affiliations:** 1 Division of Infection Medicine, Department of Clinical Sciences Lund, Lund University, Lund, Sweden; 2 Division of Dermatology and Venereology, Department of Clinical Sciences Lund, Lund University, Lund, Sweden; 3 Department of Dermatology, Skane University Hospital, Lund, Sweden; 4 Department of Biomedicine, Aarhus University, Aarhus, Denmark

## Abstract

*Cutibacterium acnes* is an abundant skin commensal with several proposed mutualistic functions. A protein with strong antioxidant activity was recently identified from the *C*. *acnes* secretome. This protein, termed RoxP, facilitated aerobic bacterial growth *in vitro* and *ex vivo*. As reducing events naturally occurred outside of the bacterial cell, it was further hypothesized that RoxP could also serve to modulate redox status of human skin. The biological function of RoxP was here assessed *in vitro* and *in vivo*, through oxidatively stressed cell cultures and through protein quantification from skin affected by oxidative disease (actinic keratosis and basal cell carcinoma), respectively. 16S rDNA amplicon deep sequencing and single locus sequence typing was used to correlate bacterial prevalence to cutaneous RoxP abundances. We show that RoxP positively influence the viability of monocytes and keratinocytes exposed to oxidative stress, and that a congruent concentration decline of RoxP can be observed in skin affected by oxidative disease. Basal cell carcinoma was moreover associated with microbial dysbiosis, characterized by reduced *C*. *acnes* prevalence. *C*. *acnes*’s secretion of RoxP, an exogenous but naturally occurring antioxidant on human skin, is likely to positively influence the human host. Results furthermore attest to its prospective usability as a biopharmaceutical.

## Introduction

The age-old relationship between bacteria and the human body is vital but ambiguous. While most bacteria can be labelled either harmful or beneficial to our health, certain multifaceted species appear to be both^1^. Transcending our current definitions of “pathogen” and “commensal”, *Cutibacterium acnes* (formerly *Propionibacterium acnes*^2^), is an abundant skin colonizer thriving in the nutritious environment of sebum rich skin^3^. Albeit causing no symptoms in the majority of carriers, this Gram-positive, facultative anaerobe holds a proposed pro-inflammatory role in acne vulgaris pathogenesis^4^ and is, due to its biofilm-forming capacity^5^, frequently isolated from various orthopedic implant-associated infections^6–8^. Despite being a well-established opportunistic pathogen, there is a link between the autoimmune disorder psoriasis and low *C*. *acnes* prevalence^9,10^ indicating that its presence is also associated with mutualistic interactions^11^. Consistent with this line of reasoning, we were recently able to characterize a protein, unique to *C*. *acnes*, with a suggested role in maintaining skin redox homeostasis^12^.

An asymmetrical distribution of hair follicles, sweat glands and sebaceous glands in our skin generates an array of distinct niches that nurtures a microbiota of great diversity^3^, highly comparable to that of our intestinal microbiota^13^. While dry areas tend to harbor a relatively small bacterial load consisting mainly of *Proteobacteria*, *Flavobacteriales* or *Bacteroidetes*, more moist and sebaceous regions, like that of our face and upper trunk, typically sustain larger quantities of *Corynebacteria*, *Staphylococci* and *Propionibacteria*^3,14^. As widely recognized for our gut microbiome^15^, this resident microbiota influences immunological responses which are, although occasionally troublesome, ultimately required to maintain cutaneous health^16^. For instance, *Staphylococci* species are capable both of soothing inflamed areas and aggravating protective pro-inflammatory responses through lipoprotein modifications^17^. *S*. *epidermidis* is furthermore able to alter the composition of the surrounding microbiota by production of antimicrobial compounds, and a complex interplay subsequently arises between our skin and the numerous bacterial species it harbors. As we increase our understanding of bacterial virulence, an accumulating amount of evidence links dysbiosis to various human disease states, including metabolic disorders^18^, atopic conditions^19^ and cancers^20^.

Combined with an emerging notion of strain-specific virulence, this challenges the concept of bacterial pathogenesis as we know it. Specific types or isolates of *C*. *acnes* can now be linked to disease^21–23^. The conventional *C*. *acnes* clades IA, IB, IC, II and III^24–26^, can be further subdivided into distinct sequence types based on single locus sequence typing^27^. These separate evolutionary lineages have repeatedly been shown to differ in their expression of virulence determinants^28–30^, including the secreted antioxidant RoxP (Radical oxygenase of *Propionibacterium acnes*)^12^. Phylotype IA strains are for example better adapted to the oxygen and fatty acid-rich environment of human skin, while phylotype IC and II strains remain closer linked to opportunistic infections of deeper tissues^31^.

Perhaps to a bigger extent than any other organ, our skin is also subjected to high quantities of reactive oxygen species (ROS) derived from ordinary metabolic reactions, cosmetics and a continuous exposure to UV irradiation from the sun. These are notorious carcinogens and have lately generated a mounting public interest in relation to antioxidant-rich food. Under physiological conditions, an endogenous production of reducing agents such as glutathione S-transferase and glutathione reductase^32^, protects our skin from excessive oxidative stress. Under pathological conditions however, the regeneration of ROS may become too great and consequently contribute to the pathogenesis of for example psoriasis^33^ or skin cancer^34^. In analogy to the production of antioxidants in human cells, evolution in this hostile milieu has likely favored the selection for several reducing agents within *C*. *acnes*^35,36^. When the bacterial secretome was first published in 2010, it disclosed a previously unidentified protein expressed in abundance by all *C*. *acnes* isolates^37^. This protein, RoxP, was later shown to exhibit high antioxidant activity^12^. RoxP proved imperative for efficient bacterial growth in aerobic settings *in vitro*_,_ as well as for the colonization of human skin *ex vivo*, ultimately raising the question as to whether it might have a corresponding role *in vivo*.

Here, we describe phylotype-specific *roxP* expression patterns and characterize RoxP activity on a molecular level. We investigate its biological function *in vitro* through human monocyte/keratinocyte cell cultures, and *in vivo* through quantification of cutaneous RoxP in oxidative skin diseases (actinic keratosis and basal cell carcinoma^38^). The potential role of *C*. *acnes* and RoxP in limiting susceptibility towards oxidative skin disease is discussed.

## Methods

### Bacterial strains and cultivation

*C*. *acnes* isolates, representative of type I, II and III strains, were grown at 37 °C under anaerobic conditions in Wilkins-Chalgren anaerobe broth (WC) until reaching either exponential (1–3 days) or stationary phase (5–7 days). Further information about specific *C*. *acnes* strains^5^ or their respective RoxP homologs^12^ can be found elsewhere.

### qPCR analysis of *roxP* gene expression

The RNA content of exponential and/or stationary phase *C*. *acnes* cultures was extracted using the RNeasy® Mini Kit (Qiagen) complemented with Bacteria Protect Reagent (Qiagen), following the manufacturer’s instructions. Resulting RNA concentrations were measured using NanoDrop® (Saveen Werner) and samples stored in −20 °C until later use.

The relative abundance of *roxP* and *gapdh* transcripts was determined through quantitative real-time PCR (qPCR) using Power SYBR Green RNA-to-Ct 1-step Master Mix (ThermoFisher) mixed 1:1 (v/v) with 10 ng RNA and 100 nM primers (*roxP*: 5′-GCATCTAGCCCTCTCACCAT-3′ and 5′-CTGAGAGTCCGGTAGGTGGT-3′; *gapdh*: 5′-GCATCATGACTACCGTCCAC-3′ and 5′-CGGTGGTCTCCTTAGAGGTC-3′) in nuclease free water. Plates were run with reverse transcription for 30 min at 48 °C, 10 min at 95 °C, and 40 cycles of 15 sec at 95 °C alternating with 1 min at 60 °C, on an iCycler iQ (Bio-Rad). Results were interpreted via double delta Cq analysis, using the recorded values for *C*. *acnes* strain 266 as a reference. The experiment was run in biological and technological duplicates.

### Generation of a RoxP expressing yeast vector

A truncated version of *roxP* (amino acid residues 24–161, omitting the N-terminal signal peptide) from *C*. *acnes* strain KPA171202 was amplified (primers: 5′-GGCTGAAGCTGAATTCACACCCATCGATGAGAGCCAAC-3′ plus 5′-GATGATGATGGTCGACTCCTGCTGCGCCGTTGAGGGCGGGATCCACC-3′) to generate a *roxP* fragment containing a C-terminal GAAG linker, and cloned into the EcoRI and SalI sites of the pPICZαA vector (ThermoFisher Scientific). Sequence verified linearized plasmids were used to transfect SuperMan5 *Pichia pastoris* (Biogrammatics).

### Expression of recombinant RoxP (rRoxP)

A generated SuperMan5 *Pichia pastoris* strain harboring the formerly described vector, was aliquoted and stored at −80 °C in YPD media supplemented with 50% glycerol. For efficient expression of recombinant protein, thawed freeze cultures were grown in a Buffered Glycerol-complex medium (BM* (70%, v/v), 0.5 M potassium buffer pH 6.0 (10%, v/v), YNB (1.4%, w/v), >99% glycerol (1%, v/v), Biotin (4 × 10^−7^%, w/v)) for 24 h prior to being re-suspended in Buffered Methanol-complex medium (BM* (70%, v/v), 0.5 M potassium buffer pH 6.0 (10%, v/v), YNB (1.4%, w/v), 99.8% methanol (1.5%, v/v), Biotin (4 × 10^−7^%, w/v)). Cultures were subsequently maintained in a shaking incubator (30 °C, 180 rpm) for 5 days with the continuous induction of *roxP* achieved through addition of methanol (1%) every 24 h.

*BM: Yeast extract (1.4%, w/v), Peptone (2.9%, w/v), CAS amino acids (1.4%, w/v).

### Purification of rRoxP

Sterile filtered yeast supernatant was loaded onto nickel columns (His Gravitrap™, GE Healthcare) and eluted according to the manufacturer’s instructions, using an imidazole buffer (20 mM sodium phosphate pH 7.4, 500 mM NaCl, 500 mM imidazole). Purified protein was immediately re-buffered in sodium phosphate (20 mM, pH 7.4) by means of dialysis (MWCO: 6–8 kDa, Novagen®), after which the protein concentration was spectrophotometrically determined through NanoDrop® (Saveen Werner) and sample purity via a 15% SDS-PAGE.

### Cryogelation

The method used for cryogelation has been previously described by Ertürk *et al*.^39^. Briefly, the functional monomer *N*-Methacryloyl-l-histidine methyl ester (1 mM) and template RoxP (1 mM) were dissolved in deionized water for precomplexation. 2-hydroxyethyl methacrylate (20 mM) was simultaneously prepared (1:1 v/v). Monomer solutions were then mixed with an equal volume cross-linking agent, N,N’-Methylenebisacrylamide (final concentration 90 mM), and the reaction mixture purged with nitric oxide. Polymerization was subsequently initialized by the addition of ammonium persulfate (4.4 mM) and N,N,N′,N′-tetramethylene ethylenediamine (0.125%). The resulting solution was aliquoted into 4 × 5 ml plastic syringes (Ø = 1.3 cm) with closed lower outlets and immediately placed in 14 °C for 24 h. After being thawed, columns were initially washed with H_2_O and template protein later removed with an imidazole buffer (20 mM sodium phosphate, 500 mM NaCl, 500 mM imidazole, pH 7.4). Elution was discontinued when no RoxP could be detected in the flow through (spectrophotometrically determined at 280 nm).

### Purification of native RoxP

The ammonium sulphate (80%) protein fraction of stationary phase *C*. *acnes* AD24 was passed through a pre-equilibrated cryogel column using a peristaltic pump (1 mL/min) under continuous stirring for 1 h. Bound RoxP was eluted using imidazole buffer (20 mM sodium phosphate, 500 mM NaCl, 500 mM imidazole, pH 7.4) and the resulting protein concentration and sample purity analyzed through NanoDrop® (Saveen Werner) and a 15% SDS-PAGE, respectively.

### ABTS radical cation assays

The reduction of pre-formed ABTS radical cations in the presence of RoxP was analyzed using a spectrophotometric assay previously described by Re *et al*.^40^. 2,2′-azinobis(3-ethylbenzothiazoline-6-sulfonic acid) diammonium salt (ABTS) (Sigma –Aldrich) was initially dissolved in ddH_2_O to a concentration of 7 nM. Potassium persulfate (MERCK) was subsequently added to a final concentration of 2.45 mM and the resulting solution kept dark and incubated overnight at RT. Formed ABTS radicals were diluted with PBS (pH 7.4) until reaching an absorbance of approximately 0.7 at 734 nm. Antioxidants (1.7 μM) were added (1:9 v/v) and the absorbance spectrum 500 to 900 nm recorded after 30 sec.

### Cell culturing

Human keratinocytes (HaCat cells) were grown in SFM medium supplemented with L-glutamine, EGF (epidermal growth factor) and bovine pituitary extract (Gibson, Invitrogen) until 90% confluency in 96-well plates (Nunc A/S, Roskilde, Denmark). Human monocytes (THP-1 cells, kindly provided by Dr. Arne Egesten) were cultured in RPMI 1640 medium supplemented with Glutamax-1 (Gibco®), 100 μg/ml streptomycin, 100 μg/ml penicillin, and 10% fetal calf serum (ThermoFisher Scientific). All cells were grown at 37 °C in 5% CO_2_ and 95% humidity.

### MTT assay

HaCat cells cultured to 90% confluency, or THP-1 cells (1.2 × 10^6^ cells/well) in a 96-well plate were treated with 2 mM paraquat (Sigma-Aldrich) and/or 5 μM RoxP for 24–48 h. HaCat cells were kept in their culture medium, while monocytes were transferred to a HEPES-HBSS (Hank’s balanced salt solution) buffer at pH 7.4. Viability was measured by the addition of 0.5 mg/ml 3-(4,5-demethylthiazol-2-yl)-2,5-diphenyltetrazolium bromide (MTT) for 1–5 hours. The cells were washed with PBS followed by the addition of DMSO. The samples incubated for 30 minutes and absorbance at 550 nm was measured using a Wallac 1420 Multilabel Counter (BioRad).

### Collection of skin swabs

Samples were collected between November 2016 and May 2017 at the Department of Dermatology at Lund University Hospital, Sweden. Patients (n = 54, aged between 50–70) presenting with clinically suspected actinic keratosis or superficial/nodular basal cell carcinoma of the face, neck, upper chest or shoulder regions, were asked for participation (Supplementary table 1). Individuals were swabbed (Venturi Transystem, Copan, Italy) with buffer pre-soaked swabs for 30 sec at lesional regions (4 cm²) and for 30 sec at an anatomically identical healthy site (4 cm²). Swabs were gently shaken in their liquid storage solution, stored at 4 °C o/n, and then prepared for analysis. Exclusion criteria included patients with: applied make up, newly washed face (<1 h), diabetes, cognitive impairment, other active skin disorders, ulcerated tumors, immunosuppression, ongoing/newly ended antibiotic or corticosteroid treatment (systemic or topical, < 4 weeks). The study was approved by the ethical committee in Malmö/Lund (2016/465), and in accordance with the Declaration of Helsinki. Written informed consent was obtained from all patients before enrollment.

### Analysis of RoxP concentration in skin swab samples using a capacitance biosensor

The abundance of RoxP in samples taken from either healthy volunteers or AK/BCC patients were analyzed on a capacitive biosensor, applying a molecular imprinting technique previously described by Ertürk *et al*.^41^. Accounting for any potential background, a calibration curve was initially obtained from RoxP spiked (0.14–0.71 mM) mock skin swabs (Venturi Transystem, Copan, Italy), diluted 1:100 (v/v) in sodium phosphate buffer (10 mM, pH 7.4). Cells and debris from patient swabs were subsequently removed through centrifugation (15 min, 4000 x g), supernatant diluted 1:100 (v/v) in sodium phosphate buffer, and eventually injected into the capacitive system in triplicates.

### Preparation of DNA from skin swabs

Cotton sticks with skin swabs from patients and healthy individuals were stored at 4 °C o/n. Cellular material was collected from the liquid storage buffer through centrifugation (5000 g, 10 min), and DNA extracted using Qiagen DNeasy. Quantity and quality of DNA extractions were evaluated by NanoDrop® (Saveen Werner).

### DNA sequencing and bioinformatics analysis

To determine the skin microbiome, the V1–V3 region of the 16S rRNA gene was amplified and amplicons were subjected to deep sequencing using Illumina technology, performed by Eurofins (Germany) and processed using the Mothur software v1.39.1 and the MiSeq SOP with some modifications. Initially, the paired-end reads were combined using the command “make.contig^42^”. The combined reads were then trimmed off the primer sequence and sequences containing ambiguous bases or containing longer stretched of homopolymers than eight were discharged from further analysis. The reads were afterwards aligned against the Silva reference alignment v128 and the resulting alignment was filtered so that all reads only overlapped the same region. To further reduce the number of sequencing errors, a pre-clustering step implementing a pseudo-single linkage algorithm originally developed by Huse *et al*.^43^ with the “diff” variable set to four was performed. Chimeras were removed using the build-in version of the UCHIME algorithm in Mothur^44^. Lastly, all singletons, duplicates, and triplicates were removed before final analysis to preclude inclusion of sequences from potential contamination or sequence errors that have not been detected in the previous steps. The resulting reads were then classified with the classify.seqs command using the Bayesian method and the taxonomic outline v14.51 from the Human Oral Microbiome Database (HOMD) (http://www.homd.org/) with a manual inclusion of relevant skin bacteria^45^. The confidence cut-off was set to 80%. Linear discriminant analysis (LDA) coupled with effect size measurement (LEfSe) to detect differentially abundant bacterial taxa (phyla, genera, species) between groups. The alpha values for the factorial Kruskal–Wallis and pairwise Wilcoxon tests were set to 0.05 and the LDA score threshold for discriminative features was set to 3.5. The online version of the LEfSe program was used^46^.

To determine phylotypes of the *C*. *acnes* population in skin swab samples we used the SLST approach established previously^29^. The SLST amplicons were subjected to MiSeq sequencing, performed by Eurofins (Germany). All high-quality sequence read pairs were assigned to SLST alleles; all currently known SLST alleles are accessible on www.medbac.dk/slst/pacnes.

## Results

### Expression of *roxP* is dependent on *C*. *acnes* phylotype

**Figure 1:**
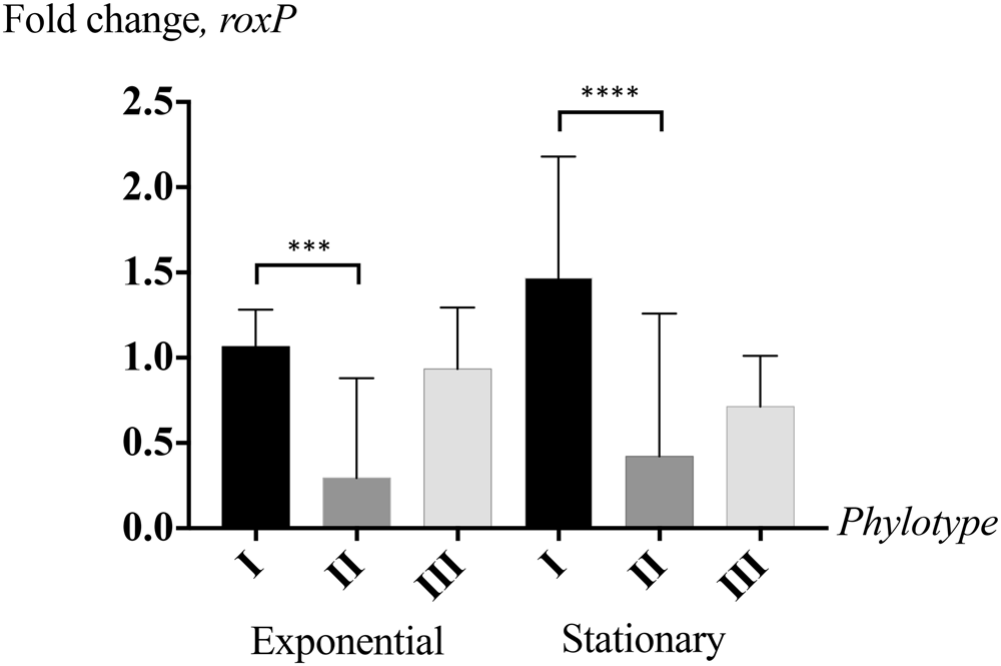
*C*. *acnes* phylotype I express significantly more *roxP* than other phylotypes, particularly during stationary phase growth. RNA was extracted from exponential and/or stationary phase *C*. *acnes* cultures, representative of type I (n = 6), type II (n = 5) and type III (n = 2) strains. qPCR quantifications of *roxP* mRNA were run in biological and technological duplicates, normalized against *gapdh* transcripts and evaluated using double delta Cq analysis. Grouped with their respective phylotypes, statistical significance was subsequently determined through a student’s t-test. ***p < 0.001, ****p < 0.0001.

Former studies have demonstrated phylotype-specific expression patterns of several *C*. *acnes* host-interacting factors^23,47^. We therefore sought to determine *roxP* expression rates through quantitative real-time PCR (qPCR) in all major phylotypes. *C*. *acnes* isolates identified as phylotype I displayed significantly higher expression levels of *roxP* compared to most phylotype II strains (Fig. 1). Sequencing of the respective *roxP* upstream regions revealed a base substitution in type II strains, affecting the - 35 region of the predicted *roxP* promoter, potentially able to negatively influence transcription rates (Fig. S1). Comparison of 155 sequenced *C*. *acnes* genomes revealed that most phylotype I strains shared the same *roxP* upstream region, associated with high expression, while the *roxP* upstream region of most phylotype II strains was distinct (Fig. S2).

As RoxP had previously proven beneficial for *C*. *acnes* regarding its oxygen tolerance^12^, we proceeded to investigate aerobic/anaerobic growth capacities of the utilized strain cohort. Whereas an increased *roxP* expression, exhibited by most phylotype I strains, correlated with an ability to grow equally well in oxic and anoxic environments, low *roxP* expression resulted in reduced or delayed growth in oxygen-rich settings (Fig. S3).

### RoxP homologs display comparable antioxidant activities

**Figure 2:**
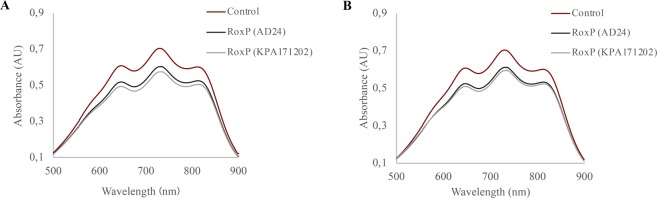
RoxP homologs display comparable activity. Two distinct RoxP variants, natively expressed from KPA171202 and AD24 *C*. *acnes*, were incubated with either (**A**) pre-formed ABTS^+^ radicals or (**B**) pre-formed ABTS^+^ radicals plus NaCl (150 mM). Reduction of substrate was measured as absorbance decrease at 734 nm.

Marked variations in *roxP* expression levels between different *C*. *acnes* phylotypes compelled us to examine, and compare, the reducing activity of different RoxP homologs (Fig. S4). Although RoxP is highly conserved, with 99–100% amino acid identity in the majority (∼93%) of isolates, a subset of strains (∼7%) classified as *C*. *acnes* phylotype II and III express a more distantly related protein, with approximately 83% amino acid identity^12^. The main variant could here be successfully isolated from (type IB) *C*. *acnes* KPA171202 culture media, and the less common homolog from (type II) *C*. *acnes* AD24 culture media. The latter was purified using macroporous cryogels imprinted with recombinant RoxP (Fig. S5), after which the protein identity was verified through western blot analysis (data not shown). As demonstrated by a decreased absorbance at 734 nm, the two homologs were able to reduce the cationic ABTS^+^ substrate with equal efficiency and, in doing so, displayed highly similar antioxidant activities (Fig. 2). Although the variant more commonly expressed by phylotype I strains displayed a continuously higher activity, the difference was only minor and not statistically significant.

### Antioxidant activity of RoxP is maintained in harsh conditions

**Figure 3:**
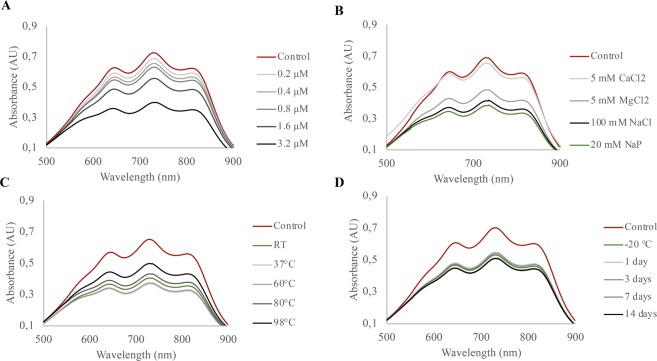
RoxP antioxidant activity is highly resilient to adverse conditions. The ability of RoxP to reduce ABTS radical cations, denoted by a decrease in absorbance at 734 nm, was tested in multiple settings. Different concentrations of recombinant RoxP (rRoxP) (0.2–3.2 μM) in sodium phosphate buffer (20 mM, pH 7.4) (**A**), rRoxP (2.7 μM) incubated with salt solutions (**B**), pre-heated rRoxP (2.7 μM) incubated for 20 min in temperatures ranging from RT to 98 °C and allowed to cool down to ambient temperature prior to activity measurements (**C**), or rRoxP (2 μM) stored at RT for up to 2 weeks (**D**) were added to a solution of pre-formed ABTS^+^ radicals and the absorbance recorded after 30 sec.

For facultative anaerobes like *C*. *acnes*, human skin presents a hostile milieu, exposing them to fluctuating temperatures, oxygen and relatively high salt concentrations^48^. In order for RoxP to possess a function *in vivo*, the protein structure would have to withstand the same diverse conditions. RoxP displayed a concentration-dependent reducing activity (Fig. 3A), had a loss of activity in presence of CaCl_2_ but withstood physiological concentrations of NaCl (Fig. 3B). The protein was furthermore able to endure temperatures of above 70 °C without any significant loss of activity (Fig. 3C), as well as long-term storage in room temperature (Fig. 3D).

### RoxP can protect human cells from oxidative damage *in vitro*

**Figure 4:**
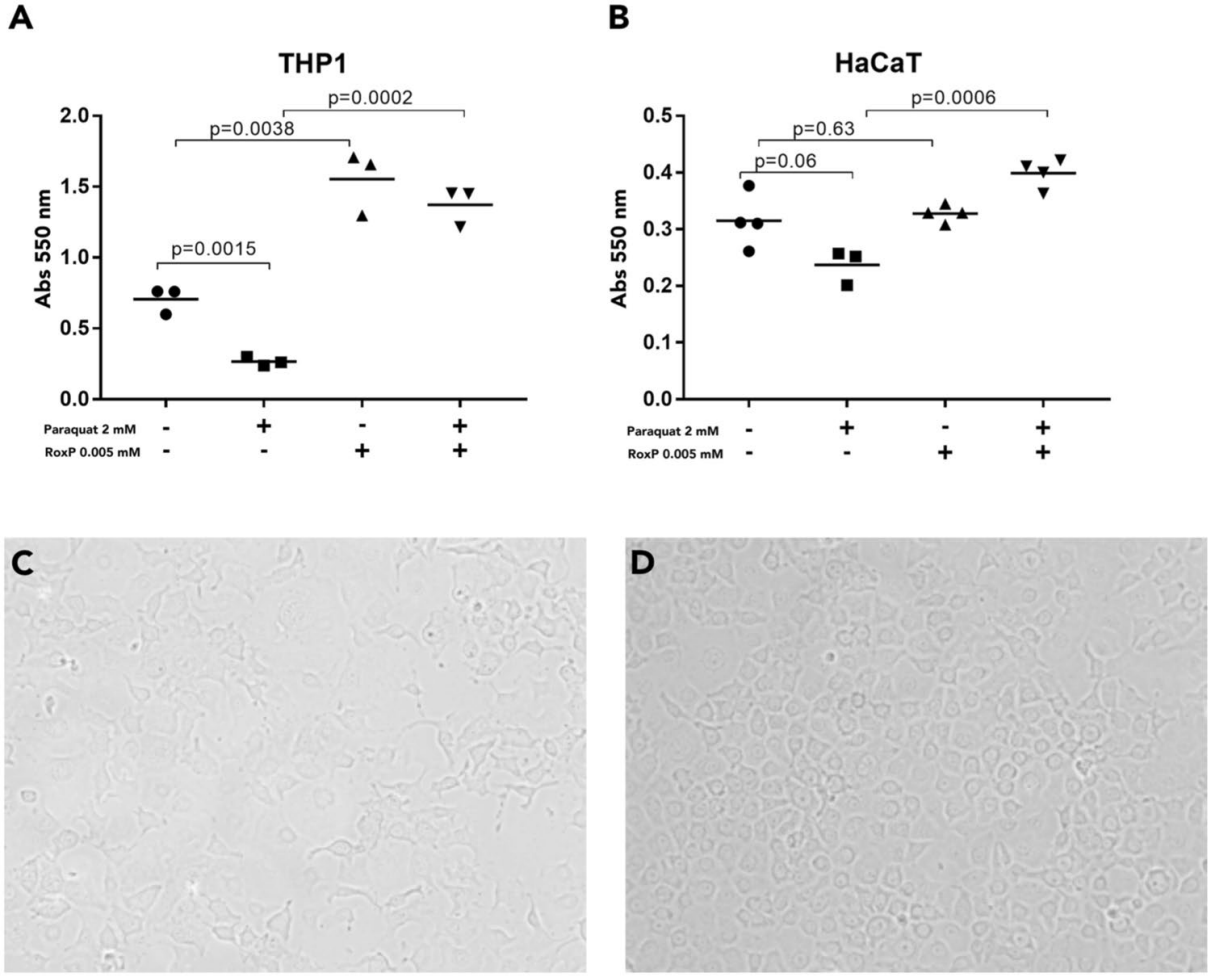
RoxP can protect human cells from oxidative damage *in vitro*. Human monocyte (**A**) or keratinocyte (**B**) cell lines were exposed to oxidative stress through addition of paraquat, while adding RoxP momentarily afterwards. Cells were incubated for 24 h (keratinocytes) or 48 h (monocytes) before cell viability was analyzed through MTT assays. Representative microscopy visualizations of monocytes after paraquat (**C**) or paraquat + RoxP (**D**) are demonstrated. Statistics were calculated using a student’s t-test.

To investigate the ability of RoxP to reduce free radicals in a biological system, we exposed HaCat and THP-1 cells to a herbicide previously shown to promote ROS generation in human cell cultures (paraquat)^49^, and studied the ability of RoxP to rescue cells from lethal oxidative stress. Even at a 1:400 ratio (RoxP:oxidizer) being added in a subsequent manner, RoxP completely inhibited the negative oxidative effect of paraquat (Fig. 4). The effect was more prominent in monocytes, while keratinocytes responded less well, both to oxidation and antioxidation (Fig. 4A,B). Notably, RoxP could also increase the overall viability of human cells in the absence of an added oxidizer (Fig. 4A).

### RoxP is less abundant in skin disease associated with oxidative stress

**Figure 5:**
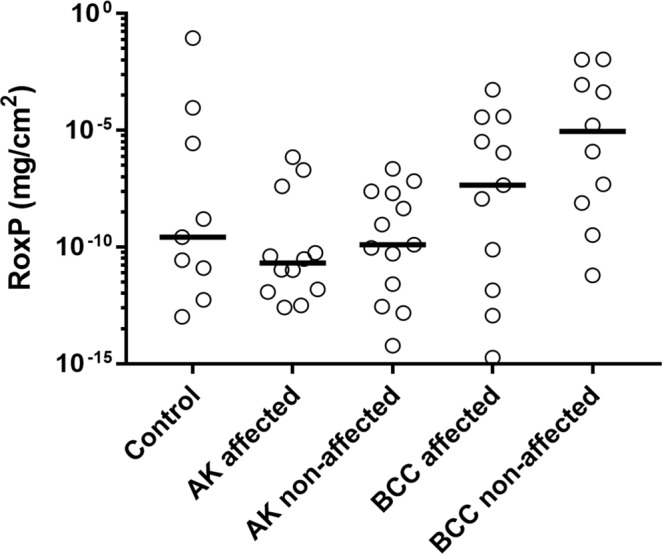
RoxP is less prevalent in skin regions with oxidative disease. Individuals with either healthy skin (control, n = 18), actinic keratosis (AK, n = 18) or basal cell carcinoma (BCC, n = 18) were swabbed at diseased (affected) and healthy (non-affected) regions. Absolute quantity of RoxP was determined by RoxP-MIP capacitive biosensor measurements. All individual data points were included in the analysis.

Oxidative stress is considered the single most influential aetiological factor in carcinogenesis of human skin^50^. Non-melanoma skin cancers, including squamous cell carcinoma and basal cell carcinoma, display not only increased evidence of oxidative stress, but also of impaired endogenous antioxidant function^51^. To investigate whether an abated function of RoxP, an exogenous but naturally occurring antioxidant, might also be linked to oxidative skin disease, we studied protein abundances in actinic keratosis (AK) and basal cell carcinoma (BCC). Skin swabs (n = 54) were obtained from individuals with healthy skin, with the pre-cancerous condition AK and with developed BCC, at both disease-affected and non-affected regions. Samples were subsequently analyzed using a highly sensitive capacitive biosensor, previously shown to detect concentrations of as little as 8.25 ng RoxP/cm^2^ skin^41^, and the recorded capacitance changes equilibrated to a pre-established calibration curve (Fig. S6). Results were analyzed on a population (Fig. 5) and individual basis (Fig. S7). In the latter case, patients were excluded if accurate determination of RoxP concentration, in either affected or non-affected regions, were unsuccessful (data not shown). An overall higher RoxP abundance was observed among patients with BCC (Fig. 5). Individuals with AK showed a RoxP concentration decline in diseased areas, while those with BCC displayed similar levels at both affected and non-affected sites (Fig. S7).

### Dysbiosis in oxidative skin disease is characterized by diminished *C*. *acnes* prevalence

**Figure 6:**
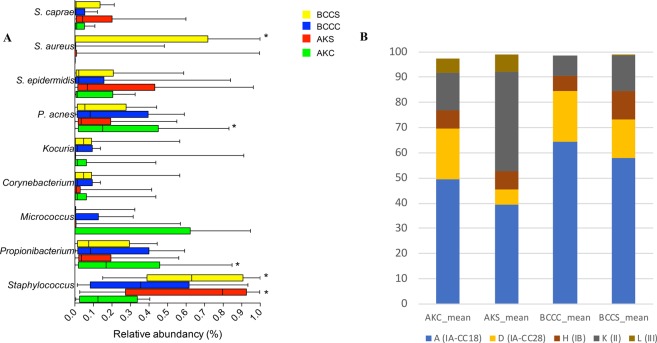
Dysbiosis of the skin microbiome in AK and BCC. Box plot of the relative abundances of the five most abundant genera and four most abundant species in the four groups (**A**). Whiskers shows the 5–95% percentile and median values are shown as vertical lines. Differentially abundant taxa based of LefSe analysis are marked with an*. (**B**) Determination of relative abundancies of *C*. *acnes* phylotypes in AK and BCC. *C*. *acnes* type II abundances increase in AK-affected skin sites compared to controls (increase from 15% to 39%), whereas type IA strains decline (from 70% to 45%).

To determine whether the observed changes to the cutaneous RoxP concentration might also have a basis in a microbial dysbiosis (e.g. *C*. *acnes*), we studied bacterial composition in AK and BCC at the genus, species and *C*. *acnes* phylotype level. Skin swabs, trailed by 16 S rDNA amplicon deep sequencing, revealed markedly diminished *Propionibacterium* and elevated *Staphylococcus* abundances in diseased compared to healthy skin (Figs 6A and S8). A drop in *C*. *acnes* prevalence during acute stages of oxidative disease (AK) seemingly proceeded the surge in staphylococcal species, particularly *S*. *aureus*, during later stages of disease (BCC) (Fig. 6A). Further characterization of *C*. *acnes* populations through single locus sequence typing (SLST) revealed that the prevalence of specific *C*. *acnes* types was also shifted in AK- and, to a lesser extent, in BCC-affected skin (Fig. 6B). Strains belonging to *C*. *acnes* type II could more commonly be isolated from diseased skin regions, while healthy skin areas displayed a closer-to-average predominance of type I strains.

## Discussion

Bacteria-derived elements directly advantageous for the human host are well-documented in the context of our gastrointestinal tract, most notably vitamin K synthesis^52,53^. Yet many of the benevolent factors identified from our cutaneous microbiome act indirectly, through colonization resistance^54,55^, rather than upon host systems themselves. Here, we study the biological effect of RoxP, a novel antioxidant secreted by the ubiquitous skin colonizer *C*. *acnes*. We show that RoxP has a direct positive influence on the viability of monocytes and keratinocytes exposed to oxidative stress *in vitro*, and that a (congruent) concentration decline of RoxP can be observed in skin affected by oxidative disease (AK) *in vivo*. We also sought to examine phylotype-specific *roxP* expression patterns and reducing activities of distinct RoxP homologs. An increased expression, presumably resulting in higher amounts of secreted, active RoxP, was noted among strains classified as *C*. *acnes* type I. These results were ultimately related to the earlier quantification of cutaneous RoxP, through analyses of prevalence and distribution of *C*. *acnes* (and other species) in AK/BCC-affected skin. Consistent with other findings, diseased skin regions displayed an overall diminished *C*. *acnes* population, with a striking decline in phylotype I strains.

Considerably higher *roxP* expression levels, indicative of enhanced oxygen tolerance, are here displayed by the skin- and acne-associated *C*. *acnes* phylotype I strains. No significant difference in reducing activity could be observed between the RoxP variants, and growth curves subsequently confirmed that elevated expression levels indeed did correlate with an overall improved aerobic growth (Fig. S3). This finding may help explain how minor genetic discrepancies between *C*. *acnes* phylotypes can impact their respective ecological preferences to such an extent^31^. Sequencing data namely suggests that a single base substitution in the promotor region of *roxP*, between *C*. *acnes* phylotype I and II (Figs S1 and S2), is influencing transcriptional activity and consequently also bacterial survival on human skin.

Activity assays were repeated under various harsh conditions, simulating *in vivo* settings. Despite being added at above physiological concentrations, sodium chloride did not significantly alter RoxP activity. The presence of calcium chloride however, almost completely abolished its radical scavenging ability; whether dependent on assay format, denaturing processes or stabilizing interactions, is not yet clear. Retaining more than 60% of its original activity after a 20 min exposure to 98 °C, RoxP furthermore demonstrated a level of thermostability seldomly observed for proteins derived from non-thermophilic organisms. The high stability of RoxP is desirable from both the aspect of using it diagnostically (biomarker) as well as a biologics. Though the exact molecular mechanism of RoxP remains to be characterized in detail, other enzymes involved in redox homeostasis (e.g. peroxidases) are notoriously heat resilient^56^, suggesting that this resistance pattern is not unique for RoxP but rather a general phenomenon for this class of proteins. Although a satisfying explanation will most likely require the elucidated three-dimensional structure, not available at present, we hypothesize that the proteins relatively minute size (∼14.6 kDa) may confer increased stability.

Further elucidating the prospective symbiotic relationship between *C*. *acnes* and its human host, the effect of RoxP was subsequently assessed in eukaryotic systems. Antioxidant addition increased the viability of monocyte/keratinocyte cell lines exposed to oxidative stress *in vitro*, even beyond that of the non-stressed sample. In doing so, RoxP seemingly rescued them from intrinsic stressors, most likely an immunological/metabolic based ROS production. If later confirmed *in vivo*, this could rationalize an involvement of *C*. *acnes* in the redox status of human skin and explain the observed prevalence decline in skin affected by oxidative disease.

The milder reaction to RoxP supplementation observed for the HaCat cell line, could theoretically be explained by an aberrant NF-*κ*B response^57^, as the pro-inflammatory NF-*κ*B signaling pathway regulates cellular responses to oxidative stress^58^. If so, this would furthermore indicate that RoxP display evidence of immunomodulatory functions, potentially exploitable in the pharmaceutical industry. Even though the concentration used (5 μM) is greater than those previously detected on skin (8.25 ng RoxP/cm^2^^41^,), it remains considerably lower than that of other antioxidants used in current skin products^59^, and is in no way immoderate. Additional studies are however required to determine whether RoxP indeed moderates NF-*κ*B or other signaling pathways.

To further assess the potential influence of RoxP activity on human skin *in vivo*, swabs were obtained from skin affected by either the pre-cancerous condition AK or the further progressed disease state BCC. Samples were analyzed for individual (diseased vs. healthy skin) dissimilarities. Closely linked to oxidative stress^50,60^, AK-affected skin regions contained lower RoxP concentrations than healthy, anatomically equivalent, sites. This may indicate a predisposed vulnerability towards physiological/pathological oxidative stress in certain areas, however, the results provide no information about causality. Reduced RoxP concentrations could be either a trigger for, or result of, disease. Samples were also analyzed for population wide (patients vs. healthy individuals) differences. Results were now indicative of an overall decreased and increased RoxP abundance among AK and BCC patients, respectively. As it has been argued that antioxidant activity may aid tumor growth and metastasis^61^, we hypothesize that RoxP (with other cutaneous antioxidants) impede tumor formation, but facilitate survival of the cancer once formed. The cutaneous microbiota could subsequently be involved in the skin’s pathophysiological events through a ROS-scavenging activity that is both preventative and abetting of cancerous conditions. It should be noted however, that results failed to demonstrate statistical significance, potentially due to big variations in composition of the individual and local microbiota. Previously shown to detect RoxP quantities of less than 9 ng RoxP/cm^2^^41^, capacitive sensors are furthermore highly sensitive instruments, prone to generate high standard deviations particularly during detection of samples including more abundant proteins (see Fig. S6).

Trends observed following quantification of RoxP *in vivo*, were ultimately correlated to studies of the local cutaneous microbiota, also in AK/BCC-affected skin. Here, oxidative disease was linked not only to a general decrease in *Propionibacterium* and *C*. *acnes* prevalence, but more specifically also to a decline of *C*. *acnes* phylotype I strains that had previously demonstrated superiority in their expression of *roxP*. Results therefore, once again, suggested that the advantageous effect of RoxP observed in human cell cultures *in vitro*, carries weight also *in vivo*. The secretion of RoxP from *C*. *acnes* seemingly aid our endogenous antioxidant systems, acting as a second layer of prevention and/or defense against oxidative disease (including psoriasis and skin cancer). This implies a role of RoxP in protection from UV irradiation and skin-aging processes that future studies will investigate.

## Conclusion

Our work demonstrates the value of a healthy skin microbiome, in particular *C*. *acnes* due to its production of RoxP, in upholding redox homeostasis of human skin, thus protecting against development of several skin pathologies driven by oxidative stress. Succeeding studies will investigate the diagnostic and therapeutic value of this bacterial antioxidant *in vivo*.

## Supplementary information

Supplementary information
