# Supplementary material for: Common skin bacteria protect their host from oxidative stress through secreted antioxidant RoxP
Source: Sci Rep. 2019 Mar 5;9:3596. doi: 10.1038/s41598-019-40471-3 (PMC6401081; doi:10.1038/s41598-019-40471-3)
Supplement: Supplementary file 1 — Supplementary information [file 41598_2019_40471_MOESM1_ESM.pdf]

Supplementary information

Article: Common skin bacteria protect their host from oxidative stress through secreted antioxidant RoxP

Author list: Tilde Andersson[1], Gizem Ertürk Bergdahl[1], Karim Saleh[2,3], Helga Magnúsdóttir[3], Kristian Stødkilde[4], Christian Brix Folsted Andersen[4], Katarina Lundqvist[2,3], Anders Jensen[4], Holger Brüggemann[4], Rolf Lood[1#]

[1]Division of Infection Medicine, Department of Clinical Sciences Lund, Lund University, Lund, Sweden. tilde@bidevind.net, gizem.erturk_bergdahl@med.lu.se, rolf.lood@med.lu.se.
[2]Division of Dermatology and Venereology, Department of Clinical Sciences Lund, Lund University, Lund, Sweden. karim.saleh@med.lu.se, katarina.lundqvist@med.lu.se.
[3]Department of Dermatology, Skane University Hospital, Lund, Sweden. helga.magnusdottirhalfin@skane.se.
[4]Department of Biomedicine, Aarhus University, Aarhus, Denmark. kst@biomed.au.dk, cbfa@biokemi.au.dk, aj@biokemi.au.dk, brueggemann@biomed.au.dk.

[#]To whom correspondence should be addressed:
Department of Clinical Sciences Lund
Division of Infection Medicine
Biomedical Center B14
Lund University
SE-221 84 Lund
Sweden
Rolf.Lood@med.lu.se

Figures

```
                                                                                -35
09_9_type_II_K1      (1.37)AAGCCGGAAGGCAGATAAACCTCT-ACCCGGGACGCCGTCGTCTCCAACACCTTAGTGAC
ATCC_11828_type_II_K9(1.37)AAGCCGGAAGGCAGATAAACCTCT-ACCCGGGACGCCGTCGTCTCCAACACCTTAGTGAC
PMH5_type_III_L      (0.73)AAACCGAAAGGCAAACCCACCCCCTACCGGGCCTACCGTTGCCGCCGGCACGTCGATGAC
523_type_IA_G        (1.94)AAACCGAAAGACAGACCCACCCCCTACCGGACATACCGTTGCCGCCAACACTTCAATGAC
PA_12_1_L1_type_IA_A (3.11)AAACCGAAAGGCAGACCCACCCCCTACCGGGCATACCGTTGCCGCCGACACTTCGATGAC
PA_15_1_R1_type_IA_C (3.11)AAACCGAAAGGCAGACCCACCCCCTACCGGGCATACCGTTGCCGCCGACACTTCGATGAC
09_109_type_II_K5    (2.10)AAACCGAAAGGCAGACCCACCCCCTACCGGGCATACCGTTGCCGCCGACACTTCGATGAC
KPA171202_type_IB_H  (2.10)AAACCGAAAGGCAGACCCACCCCCTACCGGGCATACCGTTGCCGCCGACACTTCGATGAC
                           ** *** *** ** *   *** *  *** *     **** * * **  *** *   ****
                                        -10
09_9_type_II_K1            ACTATGCAGTGCTATGCTCCAAACGAAAGTCTAAATGATTTTCGCTAATTTCCATACGTA
ATCC_11828_type_II_K9      ACTATGCAGTGCTATGCTCCAAACGAAAGTCTAAATGATTTTCGCTAATTTCCATACGTA
PMH5_type_III_L            ACTATGCAGTGCTACGCTCTAGCAGAAAATTCATATGATTTTCGTCAATTCCCATACGTA
523_type_IA_G              ACTATGCAGTGCTACGCTCTAGTCGAGAATCTATATGATTTTCGTCAATTCCCATACGTA
PA_12_1_L1_type_IA_A       ACTATGCAGTGCTATACTTCAGCTGAAAGTCTATATGATTTTCGTCAATTCCCATACGTA
PA_15_1_R1_type_IA_C       ACTATGCAGTGCTATACTTCAGCTGAAAATCTATATGATTTTCGTCAATTCCCATACGTA
09_109_type_II_K5          ACTATGCAGTGCTACACTTCAACTGAAAATCTATATGATTTTCGTCAATTCCCATACGTA
KPA171202_type_IB_H        ACTATGCAGTGCTACACTTCAACTGAAAATCTATATGATTTTCGTCAATTCCCATACGTA
                           **************  **  *   ** * *  * **********  **** *********

09_9_type_II_K1            CGACATATCGGGAGACCATCATG
ATCC_11828_type_II_K9      CGACATATCGGGAGACCATCATG
PMH5_type_III_L            CGACATATCAGGAGATCGTCATG
523_type_IA_G              CGACATATCGGGAGATCGTCATG
PA_12_1_L1_type_IA_A       CGACATATCGGGAGATCGTCATG
PA_15_1_R1_type_IA_C       CGACATATCGGGAGATCGTCATG
09_109_type_II_K5          CGACATATCGGGAGATCGTCATG
KPA171202_type_IB_H        CGACACATCGGGAGATCGTCATG
                           ***** *** ***** * *****
```

Figure S1: Alignment of the *roxP* upstream region of eight strains. The strains represent the eight dominant *roxP* upstream sequences, covering the entire *C. acnes* population (see Figure S2). The upstream sequences contain the putative *roxP* promoter, underlined. This promoter was found using the tool BPROM (V. Solovyev and A Salamov, In Metagenomics and its *Applications in Agriculture, Biomedicine and Environmental Studies,* p. 61-78, 2011.) (the confidence values, Linear discriminant function (LDF) scores, are given in brackets after the strain name). In addition, the *C. acnes* consensus promoter sequence (-10 region: T.A.n.n.n.T and -35 region: G/A.n.T/G.T/G.n.G), identified by Lin *et al.* (Lin YF, A DR, Guan S, Mamanova L and McDowall KJ, In *BMC Genomics.* Sep 14;14:620, 2013), was compared; all matching bases are highlighted in green. Type IA strains with the SLST type A and C carry a *roxP* upstream sequence with a perfect match to the consensus promoter. It is apparent that type II strains represented by strains 09-9 and ATCC 11828 carry mismatches in the -35 region.

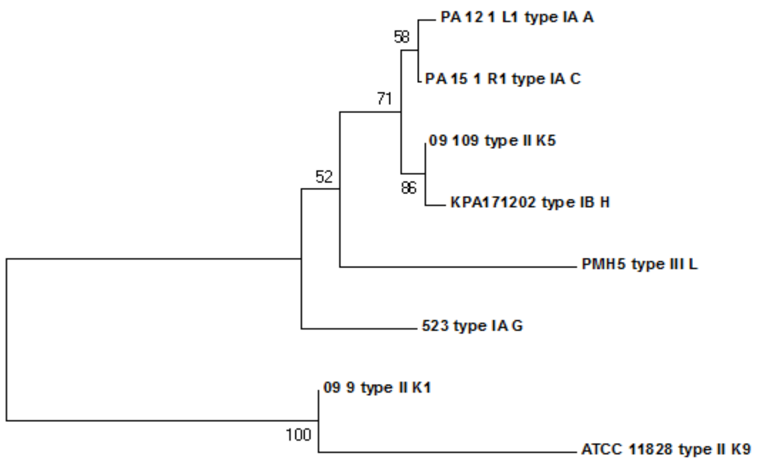

Figure S2: Analysis of the *roxP* upstream region containing the putative promoter. 155 sequenced *C. acnes* genomes were screened for the *roxP* upstream region (200 nt). All *C. acnes* genomes carried this region. Eight dominant sequences were found; representative strains carrying each an individual *roxP* upstream sequence are shown. The respective upstream regions were phylogenetically compared. Most sequenced genomes carried the version represented by strain PA_12.1.L1: 81 genomes; the other strains represented: PA_15.1.R1, 24 genomes; 09-109, 8 genomes; KPA171202, 11 genomes; PMH5, 4 genomes; 523, 3 genomes; 09-9, 2 genomes; ATCC_11828, 20 genomes. The respective type names IA, IB, II, III as well as the SLST types A, C, G, H, K, L of the strains are given in the figure. Please notice that type II strains can be found in different clusters.

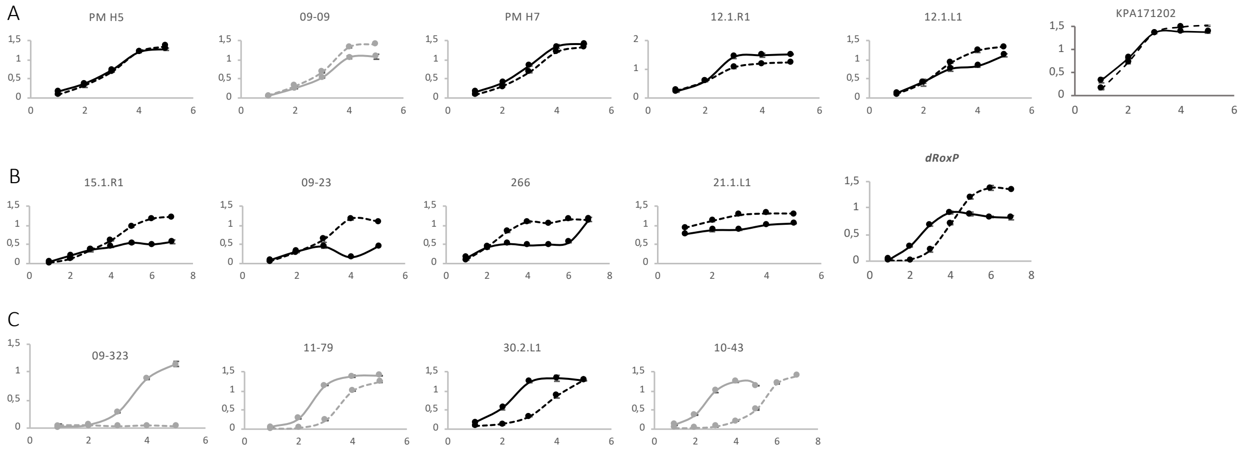

Figure S3. Tolerance to oxic conditions is strain- and RoxP- dependent in *C. acnes*. Fifteen different strains of C. *acnes* were cultured in TSB under anaerobic (solid line) or aerobic (dashed line) conditions for 5-7 days. Certain strains **(A)** showed no significant difference between oxic and anoxic growth, **(B)** were favoring aerobic growth mainly in the late exponential phase, or **(C)** had an increased lag time in aerobic conditions. Lines colored in black represent strains expressing the most common RoxP homologue, and gray lines represent strains expressing the less common protein variant. The Y-axis describes OD_{600} and the X-axis represents time (days).

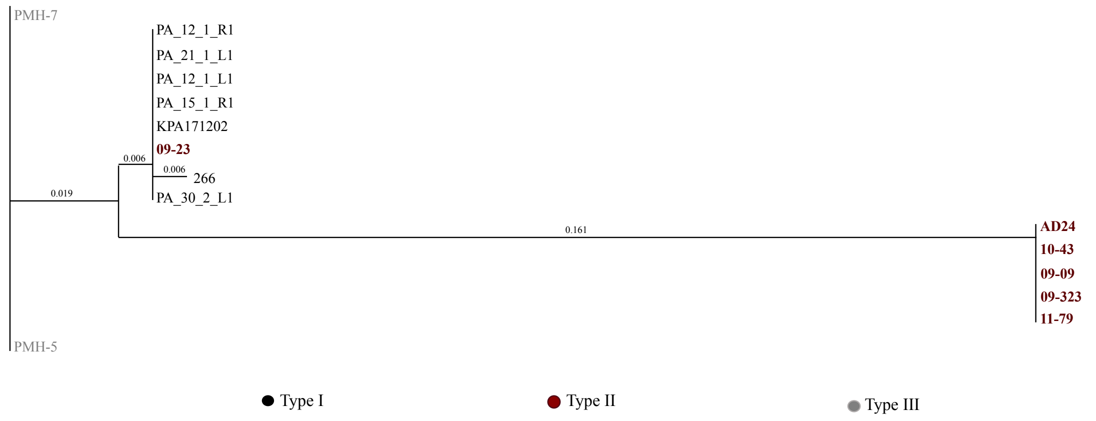

Figure S4. Phylogenetic tree of RoxP sequences from *C. acnes* separates RoxP into three main types. Amino acid sequences of RoxP from the investigated *C. acnes* strains were aligned using a ClustalW algorithm. Strains were colored based on their *C. acnes* phylotype correspondence (e.g. type I-III).

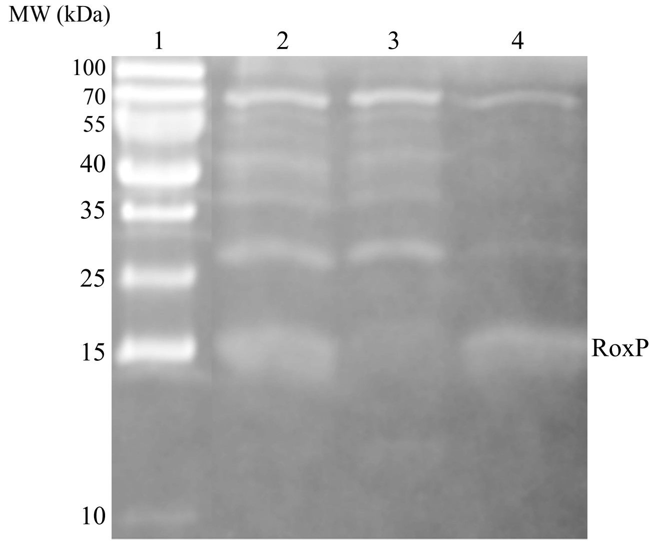

Figure S5. RoxP-based molecular imprint cryogel matrix can affinity-purify RoxP from bacterial supernatant to high purity. Ammonium sulphate precipitated (80%) and re-buffered (100 mM sodium phosphate pH 7.0) supernatant from *C. acnes* type II strain AD24 (lane 2) were run over a RoxP-based molecular imprint cryogel matrix. The flow through was collected (lane 3), and bound proteins eluted with 500 mM imidazole (lane 4). PageRuler Prestained Protein Ladder was used as a molecular weight determinant (lane 1).

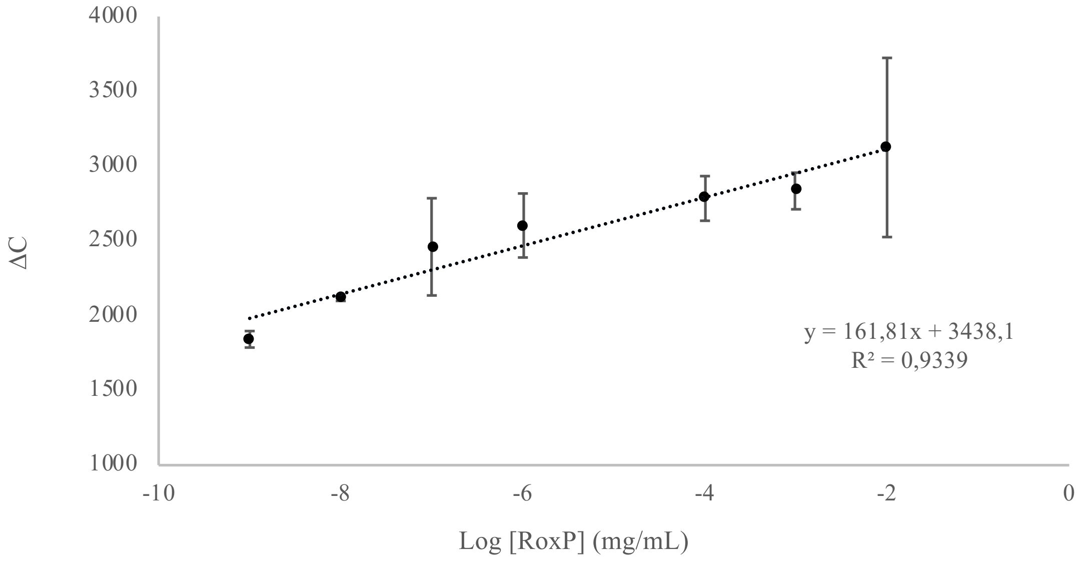

Figure S6. Calibration curve for determination of RoxP concentrations in skin swab samples using a capacitive biosensor. The contents of aerated skin swabs, not applied to skin or other solid surfaces, was diluted in a 10 mM sodium phosphate buffer pH 7.0 supplemented with ranging concentrations of RoxP. Samples were injected into the system and the resulting change in capacitance plotted against logarithmic protein concentrations.

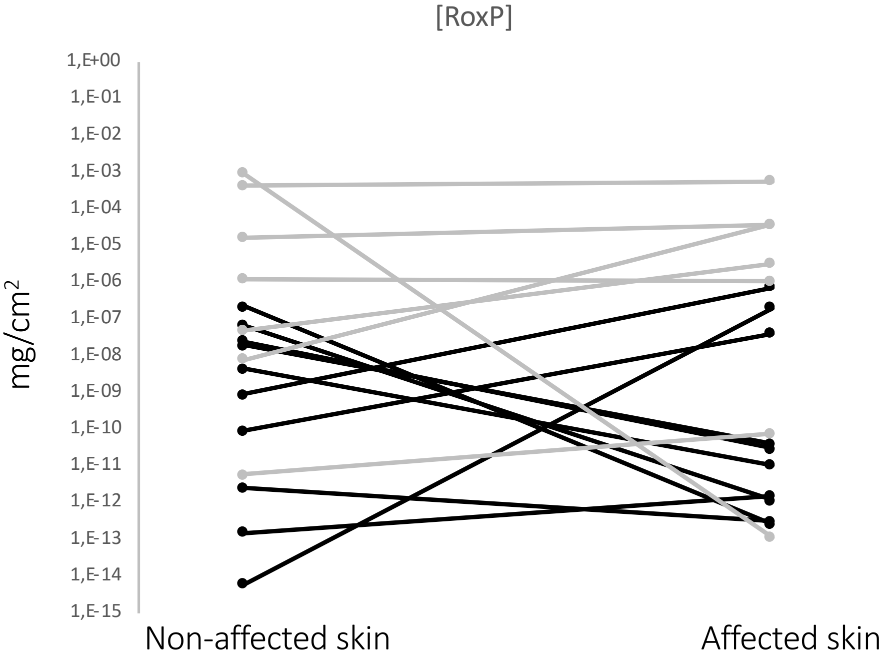

Figure S7. RoxP is prevalent in patients with BCC. Individuals with either basal cell carcinoma (grey) or actinic keratosis (black) were swabbed at diseased (affected) and healthy (non-affected) regions of the skin. Absolute quantity of RoxP was determined by RoxP-MIP capacitive biosensor measurements. The analysis includes only those individuals from whom two matching data points could be successfully computed.

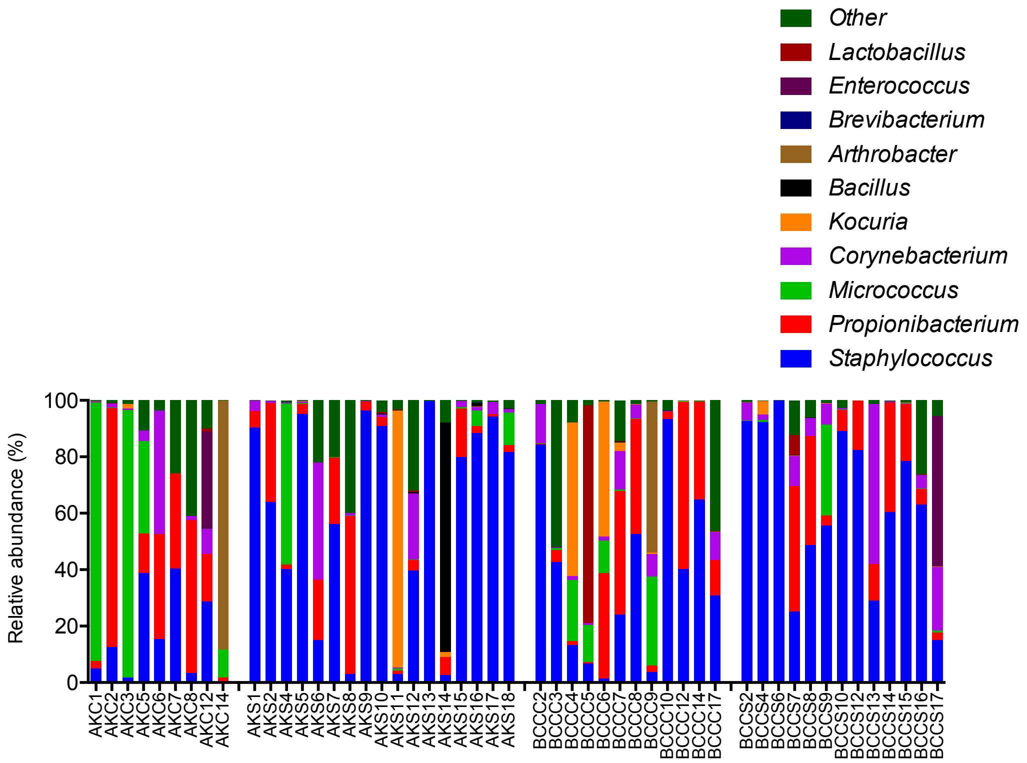

Figure S8. Dysbiosis of the skin microbiome in AK and BCC on individual patient level. Relative abundance of the ten most abundant genera (all other genera clustered in the "Other" group) in the four groups: AKC (actinic keratosis: healthy skin site), AKS (actinic keratosis: affected skin site), BCCC (basal cell carcinoma: healthy skin site), and BCCS (basal cell carcinoma: affected skin site).

Table S1. Patient characteristics and selected baseline values. Patient groups were compared using a one-way ANOVA test.

	Control	AK	BCC	*P* value
Age				.058
Range	51-70	53-69	50-70	
Mean ± SD	61.56 ± 4.58	60.5 ± 5.52	64.61 ± 5.51	
Median	61	60.5	66.5	
Sex, n				.932
Male	9	8	8	
Female	9	10	10	
Swab location				**.010***
Scalp	0	1	0	
Forehead	0	3	1	
Eyebrow	0	4	0	
Temple	0	1	2	
Nose	0	0	5	
Cheek	18	7	4	
Lip	0	1	0	
Chest	0	0	2	
Shoulder	0	1	4	

AK: Actinic keratosis. BCC: Basal cell carcinoma. * Statistically significant.
